# β Band Rhythms Influence Reaction Times

**DOI:** 10.1523/ENEURO.0473-22.2023

**Published:** 2023-06-27

**Authors:** Elie Rassi, Wy Ming Lin, Yi Zhang, Jill Emmerzaal, Saskia Haegens

**Affiliations:** 1 Donders Institute for Brain, Cognition, and Behaviour, Radboud University, 6525 EN Nijmegen, The Netherlands; 2 Department of Psychology, Centre for Cognitive Neuroscience, Paris-Lodron-University of Salzburg, 5020 Salzburg, Austria; 3 Hector Research Institute for Education Sciences and Psychology, University of Tübingen, 72074 Tübingen, Germany; 4 Department of Psychiatry, Columbia University, New York, NY 10032; 5 Human Movement Biomechanics Research Group, Department of Movement Sciences, Katholieke Universiteit Leuven, B-3001 Leuven, Belgium; 6 REVAL Rehabilitation Research Centre, Faculty of Rehabilitation Sciences, Hasselt University, 3500 Diepenbeek, Belgium; 7 Division of Systems Neuroscience, New York State Psychiatric Institute, New York, NY 10032

**Keywords:** auditory discrimination, β rhythms, frequency shift, magnetoencephalography, oscillations

## Abstract

Despite their involvement in many cognitive functions, β oscillations are among the least understood brain rhythms. Reports on whether the functional role of β is primarily inhibitory or excitatory have been contradictory. Our framework attempts to reconcile these findings and proposes that several β rhythms co-exist at different frequencies. β Frequency shifts and their potential influence on behavior have thus far received little attention. In this human magnetoencephalography (MEG) experiment, we asked whether changes in β power or frequency in auditory cortex and motor cortex influence behavior (reaction times) during an auditory sweep discrimination task. We found that in motor cortex, increased β power slowed down responses, while in auditory cortex, increased β frequency slowed down responses. We further characterized β as transient burst events with distinct spectro-temporal profiles influencing reaction times. Finally, we found that increased motor-to-auditory β connectivity also slowed down responses. In sum, β power, frequency, bursting properties, cortical focus, and connectivity profile all influenced behavioral outcomes. Our results imply that the study of β oscillations requires caution as β dynamics are multifaceted phenomena, and that several dynamics must be taken into account to reconcile mixed findings in the literature.

## Significance Statement

Spontaneous changes in brain rhythms can bias performance on perceptual tasks. Here we focus on human beta band rhythms (∼13–30 Hz) and find that not only their power, but also their frequency are related to reaction times. We observe different effects in sensory and motor cortices, suggesting there could be multiple dynamics by which beta rhythms influence behavior.

## Introduction

β Rhythms (∼13–30 Hz) are traditionally associated with the sensorimotor system where they are prominent (Pfurtscheller and Lopes da Silva, 1999). Beyond this sensorimotor role, β has been implicated in a wide range of cognitive phenomena including visual perception (Piantoni et al., 2010; Kloosterman et al., 2015), language processing (Weiss and Mueller, 2012), working memory (Axmacher et al., 2008; Siegel et al., 2009), long-term memory (Hanslmayr et al., 2016), decision-making (Wimmer et al., 2016; Wong et al., 2016), and reward processing (Marco-Pallarés et al., 2015). In nonhuman primates, β was shown to reflect top-down attention (Buschman and Miller, 2007), and in rodents, β was linked to working memory (Parnaudeau et al., 2013; Bolkan et al., 2017). However, the functional role of β is still unclear (Engel and Fries, 2010; Kilavik et al., 2012), as some studies report decreased β with task engagement (Hanslmayr et al., 2009), suggesting an inhibitory function, while others report the opposite (Kornblith et al., 2016), suggesting an excitatory function. Similarly, on the neural level, there have been mixed and contradictory findings on the relationship between β and other neural measures such as firing rate (Rule et al., 2017) and blood oxygenation level-dependent (BOLD) activity (Hanslmayr et al., 2011).

Current accounts of β mechanism and function have tried to reconcile these findings (Engel and Fries, 2010; Spitzer and Haegens, 2017). One account states that β band activity is related to the maintenance of the current sensorimotor or cognitive state via a top-down mechanism (Engel and Fries, 2010). Our account suggests that β band activity is involved in (re)activating latent sensorimotor and cognitive states (Spitzer and Haegens, 2017). We further propose that several β rhythms co-exist, including functionally inhibitory β as predominantly observed in sensorimotor regions, and functionally excitatory β as observed throughout cortex. These different β rhythms possibly operate at different frequencies (Spitzer and Haegens, 2017). At the neurophysiological level, we posit that while β events are likely excitatory in nature, there are several biologically plausible ways they could lead to functional inhibition, for example by activating inhibitory neurons or saturating excitatory neurons (Shin et al., 2017; Spitzer and Haegens, 2017).

β Activity has been characterized and modeled as transient, high-amplitude events or “bursts,” which can be detected at the single-trial level (Lundqvist et al., 2016; Sherman et al., 2016). β Bursts have been observed both focally (Bonaiuto et al., 2021) and as part of long-range communication between brain regions, where β band synchrony is assumed to facilitate interareal connectivity (Seedat et al., 2020). One property of β that has received little attention is instantaneous variability in its peak frequency (Cohen, 2014). Here, we asked how frequency shifts within the β band influence behavior.

The influence of β on behavioral outcomes might depend on several factors such as β power, frequency, bursting properties, cortical focus, and connectivity profile. In the current experiment we investigated the relationship between single-trial β activity and behavior, specifically reaction times. Since analyzing neural activity in a prestimulus or pretarget interval is a convenient method to uncover the influence of ongoing neural activity on subsequent behavior (Rassi et al., 2019b), we made use of magnetoencephalography (MEG) data recorded during an auditory sweep discrimination task. To test how the various characteristics of β relate to behavior, we analyzed reaction times as a function of pretarget β differences in power, shifts in frequency, and bursting profiles, within and between motor and auditory cortices.

## Materials and Methods

### Participants

We recorded MEG in 35 adult participants, 28 of which we included in our analyses (22 female; mean age = 22.86 years, SD = 2.84; three participants excluded because of excessively noisy MEG data and four because of near-chance performance on the task). The study was approved by the local ethics committee. All participants gave informed consent before the experiment and were given monetary compensation for their participation.

### Auditory target discrimination task

**Figure 1.**
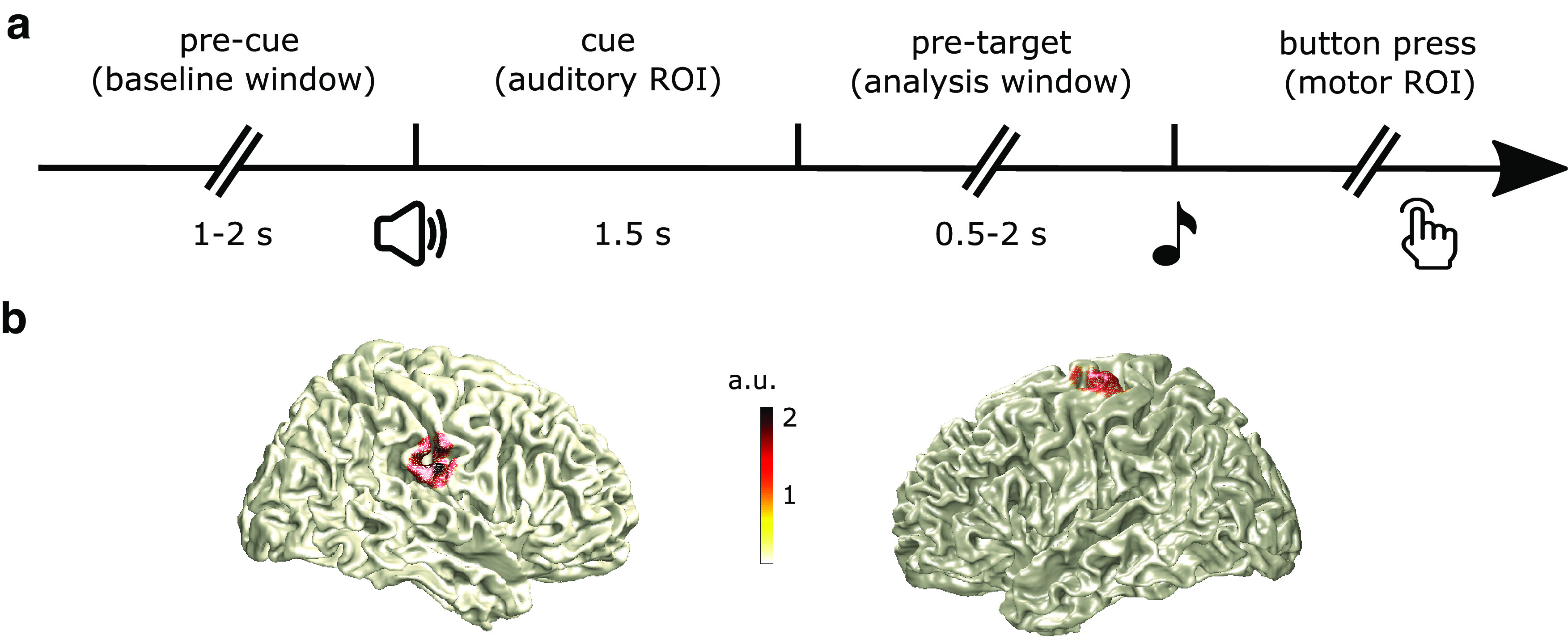
Trial sequence and region of interest definition. ***a***, After a variable baseline delay (1–2 s), an auditory cue lasting 1.5 s played, followed by a variable pretarget delay (0.5–2 s). This pretarget delay was our analysis window. After target onset, participants responded as fast as possible, indicating via button press whether the target tone shifted upward or downward in pitch. ***b***, Regions of interest (ROIs) were defined as the source location with maximum evoked activation versus baseline, based on the evoked response to the auditory cue for the auditory cortex ROI (left panel) and based on the evoked response to the button press for the motor cortex ROI (right). Showing source reconstruction for one representative subject (with a 95%-maximum activity threshold applied for illustrative purposes).

The auditory target discrimination task consisted of five rhythmic blocks and five nonrhythmic blocks (60 trials per block). The order of the blocks was randomized. In the rhythmic blocks, four cue tones were presented, separated by 0.5 s. Following the rhythmic cue, a target tone was presented at 0.5, 1, 1.5, or 2 s (80% of trials) or at 0.75, 1.25, 1.75 s (20% of trials) after the onset of the last cue tone. In the nonrhythmic blocks, the cue tone was presented continuously for a period of 1.5 s, followed by a target that was presented with a flat probability distribution within a window of 0.5–2 s after cue offset. The task for the participant was to determine whether the target tone (a 40-ms chirp) went up or down in pitch. As we had previously shown the experimental cueing manipulation not to produce behaviorally different effects (Wilsch et al., 2020; Lin et al., 2021), here we pooled all trial types (Fig. 1*a*).

### Stimuli

The cue tones had a pitch frequency of 400 Hz, a sample rate of 44,100 Hz, and a duration of 40 ms (rhythmic blocks) or 1.5 s (nonrhythmic blocks). We used a Hanning taper to remove sharp edges. The target tone consisted of 30 different frequencies randomly drawn from within 500–1500 Hz. The target was a frequency-modulated sweep created with the MATLAB function *chirp* and was either increasing or decreasing in pitch. The sound had a 10-ms cosine ramp fading in and fading out to avoid onset and offset click perception. The resulting target tone had a sample rate of 44,100 Hz and a duration of 40 ms.

We normalized all sounds (using peak normalization) to the same sound pressure level. We individually adjusted target stimuli to participants’ discrimination threshold, using a custom adaptive-tracking procedure aiming for a discrimination performance between 65% and 85% correct responses. The threshold was the slope of the pitch increase and decrease, measured as the range from lowest to highest frequency (starting point to end). We presented each participant with a pair of sounds (“up” and “down”) consisting of the a priori randomly generated frequencies, modulated depending on their individual threshold, i.e., the 30 base-frequencies were the same for each participant, with the participant’s individual threshold changing the start and end frequencies of the sounds.

### Data acquisition

Whole-head MEG data were recorded at a 1200-Hz sampling rate with a 275-channel CTF MEG system with axial gradiometers (CTF MEG Systems, VSM MedTech Ltd.) in a magnetically shielded room. To monitor the participants’ head movements online and for offline co-registration of anatomic landmarks, three fiducial coils were placed at the nasion and both ear canals. Anatomical, T1-weighted MRI scans for source localization purposes were obtained in a separate session, using either a 1.5 or 3 T Siemens MRI system (Siemens). To co-register the MEG and MRI data, we additionally mapped the scalp with Polhemus 3D (Polhemus).

### MEG and MRI preprocessing

We processed the MEG data offline with the Fieldtrip toolbox (Oostenveld et al., 2011). First, we down-sampled the data to a sampling frequency of 300 Hz. We then applied a notch filter at 50 Hz to remove line noise. Next, we segmented trials into 6-s segments starting 1 s before cue onset. We rejected bad channels (∼5%) and bad trials (∼10%) via visual inspection before independent component analysis (runICA as implemented in Fieldtrip), which was used to visually detect and remove components representing eye blinks and heartbeats.

We co-registered the MRI to the CTF coordinate system using the fiducial points and the mapped scalp surface, and segmented the MRI image with SPM8 (as implemented in Fieldtrip).

### MEG source reconstruction

We used the obob_ownft toolbox for source reconstruction (https://gitlab.com/obob/obob_ownft). In order to model virtual sensors at the locations of maximum evoked activity in both the auditory and motor sources in the right and left hemispheres respectively, we used a linearly constrained minimum variance (LCMV) beamformer approach (Van Veen et al., 1997). We first constructed volume conduction models of the participants’ brains using a single-shell model of their individual anatomic scans (Nolte, 2003), which we then used to compute leadfields for each of 3000 gridpoints. Using these leadfields, we computed common spatial filters for each participant using time windows that included a baseline period and the evoked responses.

For the auditory source, we used a time window of 100 ms centered at the peak of the individual auditory evoked response, time-locked to the onset of the auditory cue, and a 100-ms baseline window before cue onset. For the motor source, we used an activation time window of 100 ms centered at the peak of the individual motor response, time-locked to the button press, and a 100-ms baseline window before the activation window). We then normalized the difference of the sources of the pre and post windows and projected onto their co-registered anatomic scans (Fig. 1*b*). For visualization, we normalized each participant’s brain to Montreal Neurologic Institute space. We then identified the location of maximum pre versus post differences in auditory and motor sources in the right and left hemispheres, respectively. Using the spatial filters for these positions, we then extracted the time series for these two virtual channels.

### Data analysis

We performed all further data analysis using the Fieldtrip toolbox (Oostenveld et al., 2011) and custom MATLAB code. We time-locked the source-reconstructed signals from auditory and motor cortices to the onset of the target tones and analyzed a 700-ms pretarget interval. To counteract the 1/f effect in the data, we took the derivative of the time-series data (Thomson and Emery, 2014). Note that whether or not we removed the 1/f component had no influence on any of our results. However, β activity in the resulting flattened spectra was more visually salient, so we used those for visualization.

### Spectral power

To compare pretarget β power with baseline (i.e., the precue period) activity, and to test the relationship between reaction times and β power, we extracted 700 ms of precue and pretarget data and computed single-trial Fourier spectra (0–30 Hz) with a fast Fourier approach and a Hanning taper, padded to 2 s for a frequency resolution of 0.5 Hz. We log-transformed the single-trial power data and extracted power in the β frequency range (13–30 Hz). For the reaction time contrast, we split the pretarget data along the median reaction time, and averaged the power spectra for faster and slower reaction times. We tested for group-level differences in both contrasts with a cluster-based permutation approach (Maris and Oostenveld, 2007), clustering across β frequencies. We estimated effect sizes by calculating Cohen’s *d* based on the average difference in the data within a cluster (Meyer et al., 2021).

For time-resolved analyses, we performed time-frequency transformation based on multiplication in the frequency domain, using a sliding time window of 250 ms in steps of 20 ms from −750 to +250 ms relative to target onset, in steps of 0.5 Hz between 13 and 30 Hz. We then averaged power within this window to obtain a per-region normalization factor and divided each time-frequency point by that factor. Finally, we averaged across the frequency dimension within the β band and extracted single-trial β time-courses for the 700-ms pretarget window.

To test the relationship between the β time course and reaction time, we z-scored the power values and reaction times, removed those with z-values above three and below −3, and used linear regression (reaction time = β power * slope + intercept), relating each single-trial, pretarget time point of β power with the subsequent reaction time on that trial. This provided a time course of regression slopes per participant. We then generated time-courses of slopes obtained by randomly shuffling the correspondence between power values and reaction times, and tested for group-level differences between the real and shuffled data with a cluster-based permutation approach (Maris and Oostenveld, 2007), clustering across the time dimension (−700–0 ms). We estimated effect sizes by calculating Cohen’s *d* based on the average difference in the data within a cluster (Meyer et al., 2021). We also obtained the *R*^2^ values associated with each of those regressions, took the maximum across time points per participant, and reported the average across participants.

### Burst properties

To examine β burst properties in the source-reconstructed signals, we used the time-frequency representations as described above. We computed the mean and standard deviation of power within a trial for each frequency, and marked the time-frequency points that exceeded two standard deviations above the mean and that lasted at least the duration of one cycle (defined as 1/frequency). We zoomed in on the precue and pretarget delays, and based on temporal and spectral adjacency, we clustered the marked time-frequency points into burst events. We then extracted six parameters of interest from these burst events: for each trial, we counted the number of burst events. Focusing on the event that contained the time-frequency point with the highest power, we extracted the maximum power, the time point with maximum power, the frequency with maximum power, the frequency range, and the time range. This gave us single-trial estimates of burst properties during the precue and pretarget intervals.

To contrast precue and pretarget burst properties at the group level, we used paired *t* tests, and estimated effect sizes by calculating Cohen’s *d*. To examine the relationship between reaction times and burst properties, we z-scored the burst properties in the pretarget interval and reaction times, and used linear regression analysis to relate them. To test for group-level relationships, we used paired *t* tests contrasting the regression slopes against a shuffled distribution.

### Instantaneous frequency

To investigate the time course of the peak β frequency in the source-reconstructed signals, we analyzed instantaneous frequency as detailed by Cohen (Cohen, 2014). Briefly, we band-passed the single-trial data within the β frequency range, applied the Hilbert transform, extracted the phase angle time series, took the temporal derivative, and applied ten median filters. This resulted in single-trial time-series of instantaneous frequency during the precue and pretarget interval.

To compare pretarget instantaneous frequency with baseline, and to test the relationship between pretarget β frequency and reaction times (based on median split), we used a cluster-based permutation approach (Maris and Oostenveld, 2007), and estimated effect size by calculating Cohen’s *d* based on the average difference in the data within a cluster (Meyer et al., 2021). To relate single-trial time-series of instantaneous frequency with reaction times, we used the same regression approach detailed above (see Spectral power) to obtain a time course of regression slopes, and tested them at the group level with a cluster-based permutation approach (Maris and Oostenveld, 2007).

To test the relationship between reaction time and peak frequencies in the power spectra, we averaged power spectra separately for slower and faster trials (based on median split), and detected the peaks of maximum power within the β range. We then contrasted the peaks at the group level with a paired *t* test, and estimated effect sizes by calculating Cohen’s *d*.

### Connectivity

To estimate the connectivity between auditory and motor cortices, we used the Fourier coefficients that we obtained in the spectral power analysis. As connectivity measures are not resolved on single-trials, we estimated them after splitting the data along the median reaction time. We computed the pairwise phase consistency (Vinck et al., 2010), a bias-free method of rhythmic synchronization. We also computed bi-variate, nonparametric Granger causality (Dhamala et al., 2008a,b), which gave us separate estimates of the connection strengths from motor to auditory cortex and vice versa. We finally contrasted slower versus faster trials on the group level with a cluster-based permutation approach (Maris and Oostenveld, 2007), clustering across the β band. We estimated effect sizes by calculating Cohen’s *d* based on the average difference in the data within a cluster (Meyer et al., 2021).

### Data and code availability

The data and code supporting the findings of this study can be found here: https://doi.org/10.17605/OSF.IO/4AYS5.

## Results

### Pretarget versus precue power, frequency, and burst properties

**Figure 2.**
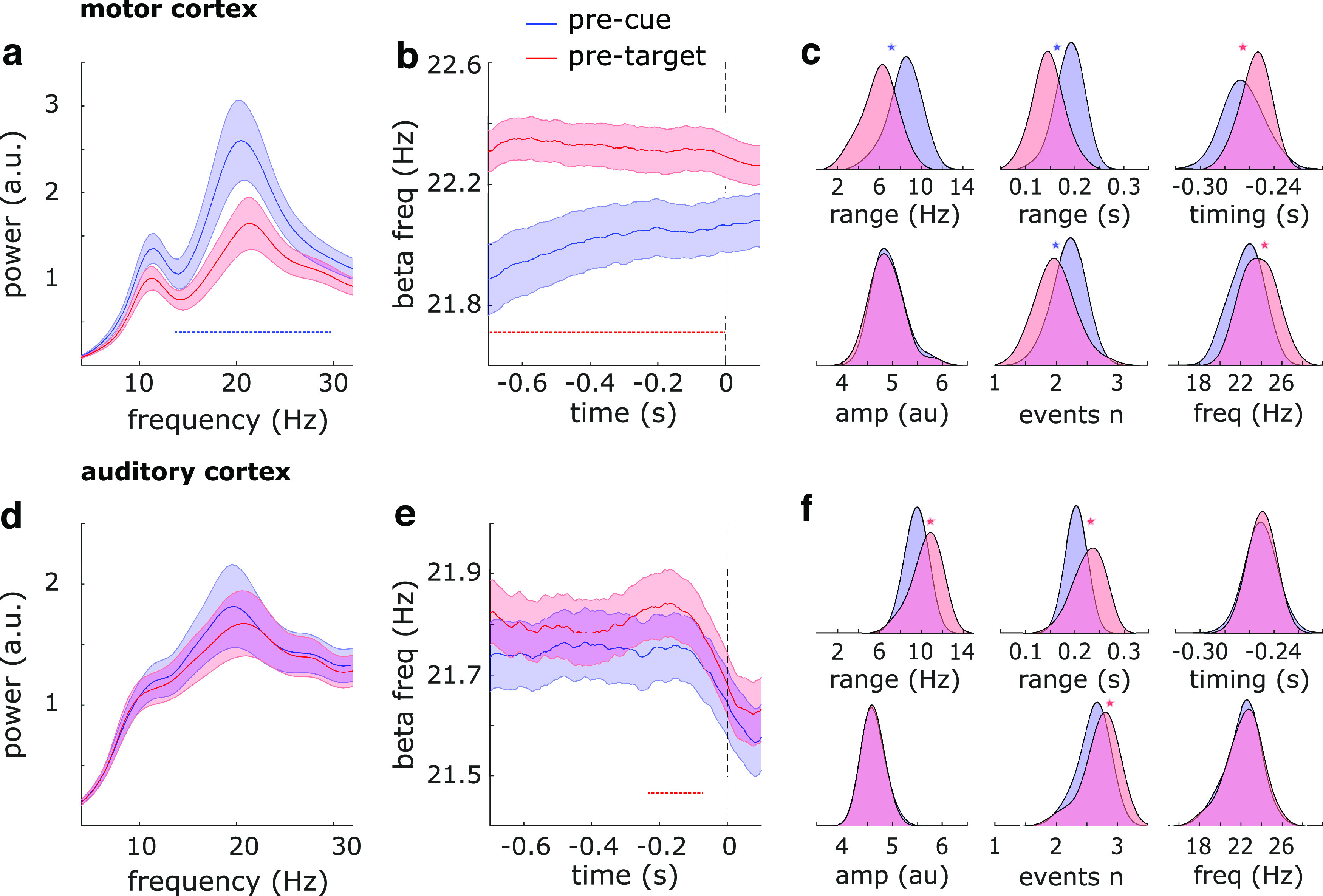
β Dynamics. ***a***, Power spectra in motor cortex during the pretarget versus precue delays. For a time-frequency representation of the same contrast, see Extended Data Figure 2-1, top. ***b***, Instantaneous β frequency in motor cortex during the pretarget versus precue delays. ***c***, β Burst properties in motor cortex during the pretarget versus precue delays: frequency range, time range, timing relative to target onset, peak amplitude, number of events, and peak frequency. ***d–f***, Same as ***a–c*** for auditory cortex. For a time-frequency representation of ***d***, see Extended Data Figure 2-1, bottom. Shaded regions around the line graphs represent the SEM. Horizontal dotted lines represent significant clusters (*p* < 0.05). Spectra in ***a*** and ***d*** were detrended by removing 1/f slope. Note that in ***b*** and ***e***, the vertical dotted lines corresponding to time point zero represent the cue onset for the precue time courses (blue) and target onset for the pretarget time courses (red). Asterisks in ***c*** and ***f*** represent significant differences between distributions.

First, we examined pretarget β properties in relation to a baseline (i.e., precue) interval (Fig. 2). We found that in motor cortex, β power decreased, and in both motor and auditory cortex, β frequency increased from baseline to pretarget interval.

10.1523/ENEURO.0473-22.2023.f2-1 Extended Data Figure 2-1 Time-frequency analysis. Top, Pretarget (vs precue) β power was significantly suppressed throughout the entire tested time-frequency range. Bottom, No significant differences in β power between pretarget and precue intervals. Download Figure 2-1, EPS file.

In motor cortex, we observed the pretarget power decrease across the whole range of β frequencies (Fig. 2*a*; cluster-based permutation test across frequencies 13–30 Hz; *p* = 1e-6, *d* = −0.78; see also Extended Data Fig. 2-1, top row, for time-frequency representations), and the upward shift in β frequency across the whole interval (Fig. 2*b*; cluster-based permutation test across time −700–0 ms; *p* = 2e-4, *d* = 0.85). Consistently, there were fewer bursts (*t*_(27)_ = −5.6, *p* = 6e-6, *d* = −1.06; Wilcoxon signed rank z-value = −3.9, *p* = 9e-5), with narrower time spans (*t*_(27)_ = −9.0, *p* = 1.3e-9, *d* = −1.70) and narrower frequency spans (*t*_(27)_ = −12.4, *p* = 1.4e-12, *d* = −2.35), and the peak burst frequency was also increased (*t*_(27)_ = 5.4, *p* = 1.3e-5, *d* = 1.01) during the pretarget delay as compared with baseline (Fig. 2*c*). In addition, bursts in motor cortex happened closer in time to target onset than they did to cue onset (*t*_(27)_ = 3.6, *p* = 0.001, *d* = 0.69). There were no differences in the maximum power of the bursts.

In auditory cortex, we observed the upward shift in β frequency primarily from 210 to 75 ms before target onset (Fig. 2*e*; *p* = 0.038, *d* = 0.52).In addition, there were more bursts (*t*_(27)_ = 5.3, *p* = 1.5e-5, *d* = 0.99; Wilcoxon signed rank z-value = 3.7, *p* = 2e-4) with wider time spans (*t*_(27)_ = 4.6, *p* = 1e-4, *d* = 0.86) and wider frequency spans (*t*_(27)_ = 5.1, *p* = 2.5e-5, *d* = 0.96) during the pretarget delay as compared with baseline (Fig. 2*f*). There were no differences in spectral power (Fig. 2*d*; Extended Data Fig. 2-1, bottom row), maximum power of the bursts, their peak frequency, or their timing relative to stimulus onset.

Next, we tested whether pretarget β properties related to reaction times using two complementary approaches: a median-split approach to relate β measures to slow versus fast reaction times, and a regression approach to relate single-trial β measures to reaction times. The two approaches yielded the same results: in motor cortex, slower reaction times were related with higher β power, while in auditory cortex, slower reaction times were related with higher β frequency.

### Spectral power

**Figure 3.**
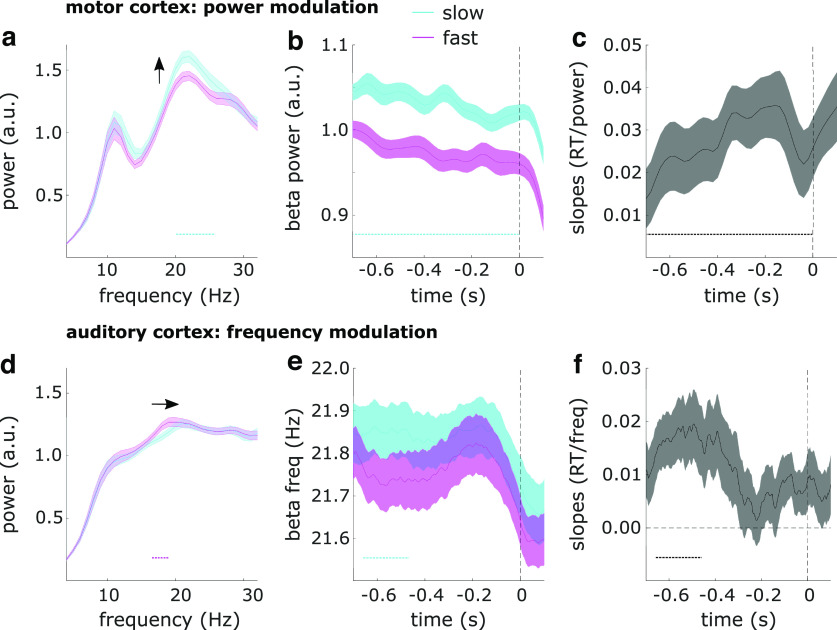
Relation between β dynamics and reaction times. ***a***, Power spectra in motor cortex for trials with slow versus fast reaction times. ***b***, Time-resolved β power in motor cortex for trials with slow versus fast reaction times. ***c***, Regression slopes for the relationship between reaction times and time-resolved β power in auditory cortex. ***d***, Same as ***a*** for auditory cortex. ***e***, Instantaneous β frequency in auditory cortex for trials with slow versus fast reaction times. ***f***, Regression slopes for the relationship between reaction times and instantaneous frequency in auditory cortex. Shaded regions around the line graphs represent the SEM. Horizontal dotted lines represent significant clusters (*p* < 0.05). Vertical dashed lines represent time point zero (target onset). Spectra in ***a*** and ***d*** were detrended by removing 1/f slope.

In motor cortex (Fig. 3*a–c*), slower reaction times were preceded by higher β power. Splitting the power spectra across the median reaction time revealed the effect was driven by differences in the 20- to 26-Hz frequency range (Fig. 3*a*; cluster-based permutation test across frequencies: *p* = 1e-5, *d* = 0.65). This effect was present throughout the whole pretarget interval when looking at the time-resolved power envelopes (Fig. 3*b*; cluster-based permutation test across time: *p* = 6e-4, *d* = 0.58). A time-resolved single-trial regression approach confirmed this effect as well (Fig. 3*c*; slope = 0.030; cluster-corrected *p* = 4e-4; *R*^2^ = 0.016). To further characterize this difference, we zoomed in on the β bursting profile and found that slower reaction times were preceded by more bursts (slope = 0.040; *p* = 0.0017) with wider time spans (slope = 0.037; *p* = 0.0277) and wider frequency spans (slope = 0.039; *p* = 0.0143).

In auditory cortex, when splitting the data along the median reaction time and contrasting the power spectra, we found an effect opposite to that observed in motor cortex, such that faster reaction times were preceded by higher β power (Fig. 3*d*; cluster-corrected *p* = 0.016, *d* = −0.63), an effect driven by differences in the 17- to 19-Hz frequency range. However, pretarget β power was not related to reaction times when using the time-resolved regression approach (slope = −0.008, no clusters). Given the discrepancy in results between the two approaches, we further investigated the observed difference in auditory cortex as a possible shift in peak frequency.

### β Frequency

In auditory cortex (Fig. 3*d–f*), slower reaction times were preceded by a higher peak β frequency when splitting the power spectra across the median reaction time and detecting participants’ individual peak β frequencies (*t*_(24)_ = 2.4, *p* = 0.026, *d* = 0.48). This effect was most pronounced around 660–450 ms before target onset when looking at instantaneous frequency (Fig. 3*e*; cluster-based permutation test across time: *p* = 0.006, *d* = 0.64). A time-resolved single-trial regression approach confirmed the same result (Fig. 3*f*; slope = 0.011; cluster-corrected *p* = 0.032; *R*^2^ = 0.019). When zooming in on the peak burst frequencies, we found the same relationship again (slope = 0.029; *p* = 0.030). In motor cortex, β frequency was not related to reaction times (slope = −0.005; no significant clusters).

### Connectivity

**Figure 4.**
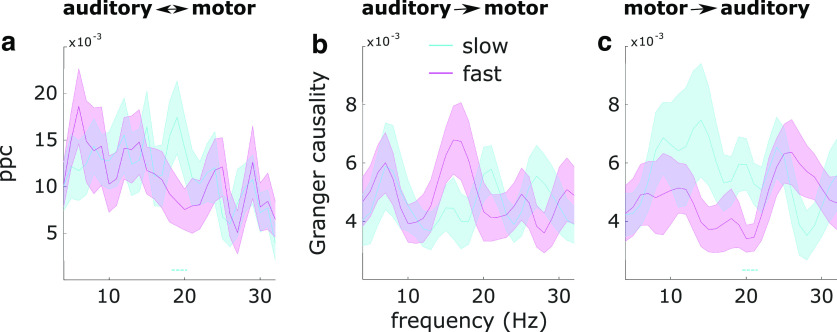
Auditory-motor cortex connectivity. ***a***, Pairwise phase consistency between auditory and motor cortices for slow versus fast reaction times. ***b***, Granger causality from auditory to motor cortex for slow versus fast reaction times. ***c***, Granger causality from motor to auditory cortex for slow versus fast reaction times. Shaded regions around the line graphs represent the SEM. Dotted lines represent significant clusters (*p* < 0.05).

We then asked whether auditory-motor β connectivity was related to reaction times (using the median-split approach). Slower reaction times were preceded by increased β connectivity between auditory and motor cortices, as quantified with pairwise phase consistency (*p* = 0.027, *d* = 0.57). The difference was most prominent at frequencies from 19 to 20 Hz (Fig. 4*a*). We then used Granger causality to check the directionality of this effect. There were no differences in auditory-to-motor β connectivity (Fig. 4*b*; no significant clusters), but slower reaction times were preceded by increased motor-to-auditory β connectivity (*p* = 0.047, *d* = 0.31), most prominently at frequencies from 20 to 21 Hz (Fig. 4*c*).

## Discussion

In an auditory target discrimination task, we sought to uncover the relationship between reaction times and various characteristics of the β rhythm. We found that slower (as compared with faster) reaction times were preceded by increased β power in motor cortex, increased β frequency in auditory cortex, and increased motor-to-auditory connectivity in the β range. The results were robust across our analysis approaches. We used a regression approach to relate single-trial reaction times to β measures, as well as a median-split approach to relate slower versus faster trials to changes in β measures, with both approaches yielding the same pattern of results. We further analyzed β activity separately in a time-resolved manner, a frequency-resolved manner, and by characterizing its burst profile, with all approaches yielding the same pattern of results.

β Activity over somato-motor cortex is traditionally viewed as a component of the μ rhythm (the other component being motor alpha activity). This β rhythm has been associated with slower movement (or lack of movement) and therefore thought to reflect cortical inhibition (Jasper and Penfield, 1949; Gastaut, 1952; Hari, 2006). More recently, β activity has been found to occur in “bursts” of high-amplitude activity, and its bursting properties have been linked to impaired performance in somatosensory perception and attention tasks (Shin et al., 2017). Our results are consistent with the literature on somato-motor β activity, as we observed increased motor β before slower responses, and we found this increased activity likely reflected an increased number of pretarget bursts with wider time and frequency ranges.

In auditory cortex, β activity has been observed in tandem with alpha activity, and assumed to serve a similarly inhibitory function (Weisz et al., 2011, 2014; Leske et al., 2014). However, in the current dataset, pretarget auditory β power was not robustly related to reaction times. This null finding precludes us from drawing decisive conclusions about auditory β power modulations. Instead, we found that an upward shift in β frequency and increased connectivity with motor cortex were related to slower reaction times. This result can be interpreted in light of the frequency-matching notion (Lowet et al., 2017). That is, on trials with slow responses, the difference in peak frequencies between auditory and motor cortex is reduced, resulting in stronger inhibitory synchronization.

Based on this set of results, we speculate that the β rhythm potentially serves different functions and operates via different mechanisms in different cortical locations. On the one hand, a local change in β power could relate to the local excitability of a brain region in an “α-like” way (Spitzer and Haegens, 2017), meaning it could reflect inhibitory processes (Klimesch et al., 2007; Jensen and Mazaheri, 2010). On the other hand, a change in β frequency could relate to β’s possible role in interareal synchrony (Bressler and Richter, 2015). For example, a shift in the frequency of β connectivity was recently shown to reflect categorical decisions (Rassi et al., 2022). It is also possible that β rhythms at different frequencies serve different functions. Although a crude distinction between “higher” and “lower” β across cortical locations has been previously made (Kopell et al., 2011), attempts to assign them different functional roles have had mixed success (Spitzer and Haegens, 2017).

The nonstationarity of β rhythm (and cortical rhythms in general) frequencies across time has so far received little attention. Models of β function account for the potential of different β rhythms occurring at different frequencies, assuming different cortical locations or different generators within a location. But it is so far under-appreciated that a single rhythm can shift in frequency over time (Cohen, 2014; Rassi et al., 2019a). Frequency shifts according to task demands have been observed in human EEG/MEG data for the alpha rhythm (Haegens et al., 2014; Samaha and Postle, 2015; Mierau et al., 2017; Wutz et al., 2018), and in nonhuman primate LFP data for the β rhythm (Kilavik et al., 2012). It has also been reported that slower α rhythms correlate with slower responses across subjects (Surwillo, 1961), but to our knowledge the relationship between β frequency and reaction times has not yet been investigated. We here report the opposite relationship for the β rhythm, such that faster (auditory) β correlated with slower reaction times within subjects.

Beyond local β dynamics, β has also been shown to be involved in long-range communication between cortical sites (Seedat et al., 2020). Here, we found increased β connectivity between motor and auditory cortex, specifically in the direction of motor to auditory cortex, before slower (vs faster) responses. It is unlikely that this effect was confounded by the power difference in motor cortex as we used a phase-based connectivity measure, and in addition, there were no robust power differences in auditory cortex. This finding is in line with the notion of covert active sensing, where the motor system actively coordinates sensory systems (Schroeder et al., 2010). In addition, oscillatory bursts have been described as channels for selective communication between brain regions, via a mechanism called frequency-division multiplexing (Akam and Kullmann, 2014). In this view, bursts at different frequencies act as distinct channels to selectively transmit neural codes to networks (regions) with appropriate filter settings that can selectively read out the codes. This interpretation links our connectivity results with the perspective that β is occurring in bursts.

Finally, our results imply that the analysis of β oscillations requires caution as β dynamics are multifaceted phenomena. For example, it is possible that observed power modulations are better explained as frequency shifts (as is the case for our results). It is also possible that the β rhythm serves different functions (i.e., inhibitory or excitatory) depending on the cortical region where it is found or depending on whether it is local or interareal. Future investigations could focus on interareal variability in β peak frequency, for example in intracranial human electrophysiological recordings.

## Synthesis

Reviewing Editor: Niko Busch, Westfalische Wilhelms-Universitat Munster Decisions are customarily a result of the Reviewing Editor and the peer reviewers coming together and discussing their recommendations until a consensus is reached. When revisions are invited, a fact-based synthesis statement explaining their decision and outlining what is needed to prepare a revision will be listed below. The following reviewer(s) agreed to reveal their identity: James Bonaiuto.

Synthesis

Both reviews are largely positive and highlight the merit of your study. However, the reviewers have a few requests for revisions, mostly regarding interpretation of the data and clarifying methodological details, and I encourage you to carefully address each of these points.

Reviewer 1

In the manuscript Beta band rhythms influence reaction times, the goal is the characterization of beta oscillations and their functional relevance. The author analysed MEG data from an auditory discrimination task (a paradigm already used in other studies from the same lab; Lin et al., 2021; Wilsch et al., 2020) focusing on 1) behavioural changes (reaction times, RT) as a function of the beta power and frequency in the auditory and motor cortex, during the pre-target vs baseline time windows. 2) The authors also perform an extensive characterisation of the Beta burst (in terms of frequency and time range, the timing of occurrence before target onset, peak amplitude, number of events and peak frequency) and their relation with RTs. 3) Finally, beta connectivity between the auditory and motor cortex was computed. The authors found that all the calculated beta indices were associated with changes in RTs. The authors discussed their findings as empirical evidence for their theoretical claim (see Spitzer and Haegens, 2017) regarding the multifaced role of the beta dynamics for behaviour.

Although the analyses performed are dense, the manuscript is very easy to read. The hypotheses are clearly stated, the method section is clear and appropriately justified, and the results are logically presented. I only have a very few points to suggest.

Although I like the conciseness of the manuscript, as the authors mentioned in the intro, beta oscillations have been underestimated for their role in affecting behaviour. Therefore, I would invite the authors to spend more time in the Discussion section and link their current data to the previously existing literature and/or theoretical accounts on the beta band more extensively. Also, it would be interesting to read the authors’ opinions, for example, on the differentiation between beta and alpha rhythms.

I would also ask the authors to add the effect size (or effect size range) for their statistics. Furthermore, where possible, can the author add the RT variance explained by the different beta metrics (e.g., beta power)?

Reviewer 2

This study presents an in-depth investigation into the relationship between beta oscillations in the brain and behavior using magnetoencephalography (MEG) during an auditory sweep discrimination task. The study found that in motor cortex, slower reaction times were preceded by higher beta power and increased motor-to-auditory beta connectivity, while in auditory cortex, slower reaction times were preceded by higher beta frequency. I found this study to be interesting and well-executed, but I have a few major comments.

Major comments:

1. I couldn’t find any information on the kernel used or the kernel width for time-frequency decomposition. It would be beneficial to see some time-frequency plots averaged over trials and for single trials. My concern is that wavelet convolution (the equivalent of frequency domain multiplication) may introduce smearing in the frequency domain due to the tradeoff between temporal and spectral resolution. This is crucial for understanding the results concerning burst peak frequency and frequency range in relation to beta instantaneous frequency.

2. Some of the apparent discrepancies between the number of bursts and changes in power of frequency could be resolved by looking at the rate of bursts with different peak amplitudes and frequencies. This could also help interpreting the results using instantaneous frequency.

3. I didn’t understand how computing the PSD from the temporal derivative of the signal would account for 1/f noise. It is unclear how this method works and if there is any literature to support this approach. Or did I misunderstand, and the derivative of the PSD was computed rather than a PSD on the temporal derivative?

4. I find it difficult to interpret the connectivity results with the perspective that beta is occurring in bursts. Were bursts more likely to be temporally aligned in motor and auditory cortex in the identified frequency range of increased motor-to-auditory connectivity?

Minor comments:

1. There was no information on the MRI scans and processing methods used

2. What type of ICA was run (fastICA)? Were the artifactual components identified visually?

3. Is there a missing reference on lines 35 -36 showing decreased beta with task engagement?

4. I think the t-test was overused in this study. For example, it doesn’t make sense to use it to compare the number of bursts events, which would have a Poisson or negative binomial distribution. It would be better to use generalized linear models with the appropriate distribution and link function. Better still to use generalized linear mixed models on all bursts with subject-specific offsets.

## References

[B1] Akam T, Kullmann DM (2014) Oscillatory multiplexing of population codes for selective communication in the mammalian brain. Nat Rev Neurosci 15:111–122. 10.1038/nrn3668 24434912 PMC4724886

[B2] Axmacher N, Schmitz DP, Wagner T, Elger CE, Fell J (2008) Interactions between medial temporal lobe, prefrontal cortex, and inferior temporal regions during visual working memory: a combined intracranial EEG and functional magnetic resonance imaging study. J Neurosci 28:7304–7312. 10.1523/JNEUROSCI.1778-08.2008 18632934 PMC6670397

[B3] Bolkan SS, Stujenske JM, Parnaudeau S, Spellman TJ, Rauffenbart C, Abbas AI, Harris AZ, Gordon JA, Kellendonk C (2017) Thalamic projections sustain prefrontal activity during working memory maintenance. Nat Neurosci 20:987–996. 10.1038/nn.4568 28481349 PMC5501395

[B4] Bonaiuto JJ, Little S, Neymotin SA, Jones SR, Barnes GR, Bestmann S (2021) Laminar dynamics of high amplitude beta bursts in human motor cortex. Neuroimage 242:118479. 10.1016/j.neuroimage.2021.118479 34407440 PMC8463839

[B5] Bressler SL, Richter CG (2015) Interareal oscillatory synchronization in top-down neocortical processing. Curr Opin Neurobiol 31:62–66. 10.1016/j.conb.2014.08.010 25217807

[B6] Buschman TJ, Miller EK (2007) Top-down versus bottom-up control of attention in the prefrontal and posterior parietal cortices. Science 315:1860–1862. 10.1126/science.1138071 17395832

[B7] Cohen MX (2014) Fluctuations in oscillation frequency control spike timing and coordinate neural networks. J Neurosci 34:8988–8998. 10.1523/JNEUROSCI.0261-14.2014 24990919 PMC6608248

[B8] Dhamala M, Rangarajan G, Ding M (2008a) Estimating Granger causality from Fourier and wavelet transforms of time series data. Phys Rev Lett 100:e018701. 10.1103/PhysRevLett.100.018701 18232831

[B9] Dhamala M, Rangarajan G, Ding M (2008b) Analyzing information flow in brain networks with nonparametric Granger causality. Neuroimage 41:354–362. 10.1016/j.neuroimage.2008.02.020 18394927 PMC2685256

[B10] Engel AK, Fries P (2010) Beta-band oscillations—signalling the status quo? Curr Opin Neurobiol 20:156–165. 10.1016/j.conb.2010.02.015 20359884

[B11] Gastaut H (1952) [Electrocorticographic study of the reactivity of Rolandic rhythm]. Rev Neurol (Paris) 87:176–182. 13014777

[B12] Haegens S, Cousijn H, Wallis G, Harrison PJ, Nobre AC (2014) Inter- and intra-individual variability in alpha peak frequency. Neuroimage 92:46–55. 10.1016/j.neuroimage.2014.01.049 24508648 PMC4013551

[B13] Hanslmayr S, Spitzer B, Bäuml KH (2009) Brain oscillations dissociate between semantic and nonsemantic encoding of episodic memories. Cereb Cortex 19:1631–1640. 10.1093/cercor/bhn197 19001457

[B14] Hanslmayr S, Volberg G, Wimber M, Raabe M, Greenlee MW, Bäuml K-HT (2011) The relationship between brain oscillations and BOLD signal during memory formation: a combined EEG-fMRI study. J Neurosci 31:15674–15680. 10.1523/JNEUROSCI.3140-11.2011 22049410 PMC6623030

[B15] Hanslmayr S, Staresina BP, Bowman H (2016) Oscillations and episodic memory: addressing the synchronization/desynchronization conundrum. Trends Neurosci 39:16–25. 10.1016/j.tins.2015.11.004 26763659 PMC4819444

[B16] Hari R (2006) Action–perception connection and the cortical mu rhythm. Prog Brain Res 159:253–260. 17071236 10.1016/S0079-6123(06)59017-X

[B17] Jasper H, Penfield W (1949) Electrocorticograms in man: effect of voluntary movement upon the electrical activity of the precentral gyrus. Arch F Psychiatr U Z Neur 183:163–174. 10.1007/BF01062488

[B18] Jensen O, Mazaheri A (2010) Shaping functional architecture by oscillatory alpha activity: gating by inhibition. Front Hum Neurosci 4:186. 10.3389/fnhum.2010.00186 21119777 PMC2990626

[B19] Kilavik BE, Ponce-Alvarez A, Trachel R, Confais J, Takerkart S, Riehle A (2012) Context-related frequency modulations of macaque motor cortical LFP beta oscillations. Cereb Cortex 22:2148–2159. 10.1093/cercor/bhr299 22021914

[B20] Klimesch W, Sauseng P, Hanslmayr S (2007) EEG alpha oscillations: the inhibition-timing hypothesis. Brain Res Rev 53:63–88. 10.1016/j.brainresrev.2006.06.003 16887192

[B21] Kloosterman NA, Meindertsma T, Hillebrand A, van Dijk BW, Lamme VAF, Donner TH (2015) Top-down modulation in human visual cortex predicts the stability of a perceptual illusion. J Neurophysiol 113:1063–1076. 10.1152/jn.00338.2014 25411458 PMC4329440

[B22] Kopell N, Whittington MA, Kramer MA (2011) Neuronal assembly dynamics in the beta1 frequency range permits short-term memory. Proc Natl Acad Sci U S A 108:3779–3784. 10.1073/pnas.1019676108 21321198 PMC3048142

[B23] Kornblith S, Buschman TJ, Miller EK (2016) Stimulus load and oscillatory activity in higher cortex. Cereb Cortex 26:3772–3784. 10.1093/cercor/bhv182 26286916 PMC5004752

[B24] Leske S, Tse A, Oosterhof NN, Hartmann T, Müller N, Keil J, Weisz N (2014) The strength of alpha and beta oscillations parametrically scale with the strength of an illusory auditory percept. Neuroimage 88:69–78. 10.1016/j.neuroimage.2013.11.014 24246486

[B25] Lin WM, Oetringer DA, Bakker-Marshall I, Emmerzaal J, Wilsch A, ElShafei HA, Rassi E, Haegens S (2021) No behavioural evidence for rhythmic facilitation of perceptual discrimination. Eur J Neurosci 55:3352–3364. 33772897 10.1111/ejn.15208 PMC9540985

[B26] Lowet E, Roberts MJ, Peter A, Gips B, De Weerd P (2017) A quantitative theory of gamma synchronization in macaque V1. Elife 6:e26642. 10.7554/eLife.26642 28857743 PMC5779232

[B27] Lundqvist M, Rose J, Herman P, Brincat SL, Buschman TJ, Miller EK (2016) Gamma and beta bursts underlie working memory. Neuron 90:152–164. 10.1016/j.neuron.2016.02.028 26996084 PMC5220584

[B28] Marco-Pallarés J, Münte TF, Rodríguez-Fornells A (2015) The role of high-frequency oscillatory activity in reward processing and learning. Neurosci Biobehav Rev 49:1–7. 10.1016/j.neubiorev.2014.11.014 25464028

[B29] Maris E, Oostenveld R (2007) Nonparametric statistical testing of EEG- and MEG-data. J Neurosci Methods 164:177–190. 10.1016/j.jneumeth.2007.03.024 17517438

[B30] Meyer M, Lamers D, Kayhan E, Hunnius S, Oostenveld R (2021) Enhancing reproducibility in developmental EEG research: BIDS, cluster-based permutation tests, and effect sizes. Dev Cogn Neurosci 52:101036. 10.1016/j.dcn.2021.101036 34801856 PMC8607163

[B31] Mierau A, Klimesch W, Lefebvre J (2017) State-dependent alpha peak frequency shifts: experimental evidence, potential mechanisms and functional implications. Neuroscience 360:146–154. 10.1016/j.neuroscience.2017.07.037 28739525

[B32] Nolte G (2003) The magnetic lead field theorem in the quasi-static approximation and its use for magnetoencephalography forward calculation in realistic volume conductors. Phys Med Biol 48:3637–3652. 10.1088/0031-9155/48/22/002 14680264

[B33] Oostenveld R, Fries P, Maris E, Schoffelen J-M (2011) FieldTrip: open source software for advanced analysis of MEG, EEG, and invasive electrophysiological data. Comput Intell Neurosci 2011:156869. 10.1155/2011/156869 21253357 PMC3021840

[B34] Parnaudeau S, O’Neill PK, Bolkan SS, Ward RD, Abbas AI, Roth BL, Balsam PD, Gordon JA, Kellendonk C (2013) Inhibition of mediodorsal thalamus disrupts thalamofrontal connectivity and cognition. Neuron 77:1151–1162. 10.1016/j.neuron.2013.01.038 23522049 PMC3629822

[B35] Pfurtscheller G, Lopes da Silva FH (1999) Event-related EEG/MEG synchronization and desynchronization: basic principles. Clin Neurophysiol 110:1842–1857. 10.1016/s1388-2457(99)00141-8 10576479

[B36] Piantoni G, Kline KA, Eagleman DM (2010) Beta oscillations correlate with the probability of perceiving rivalrous visual stimuli. J Vis 10:18. 10.1167/10.13.18 21149311

[B37] Rassi E, Dorffner G, Gruber W, Schabus M, Klimesch W (2019a) Coupling and decoupling between brain and body oscillations. Neurosci Lett 711:134401. 10.1016/j.neulet.2019.134401 31349018

[B38] Rassi E, Fuscà M, Weisz N, Demarchi G (2019b) Detecting pre-stimulus source-level effects on object perception with magnetoencephalography. J Vis Exp (149), e60120. 10.3791/60120 31403630

[B39] Rassi E, Zhang Y, Mendoza G, Mendez JC, Merchant H, Haegens S (2022) Distinct beta frequencies reflect categorical decisions. Nat Commun 14:2923. 10.1038/s41467-023-38675-3 PMC10203257 37217510

[B40] Rule ME, Vargas-Irwin CE, Donoghue JP, Truccolo W (2017) Dissociation between sustained single-neuron spiking and transient β-LFP oscillations in primate motor cortex. J Neurophysiol 117:1524–1543. 10.1152/jn.00651.2016 28100654 PMC5376602

[B41] Samaha J, Postle BR (2015) The speed of alpha-band oscillations predicts the temporal resolution of visual perception. Curr Biol 25:2985–2990. 10.1016/j.cub.2015.10.007 26526370 PMC4654641

[B42] Schroeder CE, Wilson DA, Radman T, Scharfman H, Lakatos P (2010) Dynamics of active sensing and perceptual selection. Curr Opin Neurobiol 20:172–176. 10.1016/j.conb.2010.02.010 20307966 PMC2963579

[B43] Seedat ZA, Quinn AJ, Vidaurre D, Liuzzi L, Gascoyne LE, Hunt BAE, O’Neill GC, Pakenham DO, Mullinger KJ, Morris PG, Woolrich MW, Brookes MJ (2020) The role of transient spectral ‘bursts’ in functional connectivity: a magnetoencephalography study. Neuroimage 209:116537. 10.1016/j.neuroimage.2020.116537 31935517

[B44] Sherman MA, Lee S, Law R, Haegens S, Thorn CA, Hämäläinen MS, Moore CI, Jones SR (2016) Neural mechanisms of transient neocortical beta rhythms: converging evidence from humans, computational modeling, monkeys, and mice. Proc Natl Acad Sci U S A 113:E4885–E4894. 10.1073/pnas.1604135113 27469163 PMC4995995

[B45] Shin H, Law R, Tsutsui S, Moore CI, Jones SR (2017) The rate of transient beta frequency events predicts behavior across tasks and species. Elife 6:e29086. 10.7554/eLife.29086 29106374 PMC5683757

[B46] Siegel M, Warden MR, Miller EK (2009) Phase-dependent neuronal coding of objects in short-term memory. Proc Natl Acad Sci U S A 106:21341–21346. 10.1073/pnas.0908193106 19926847 PMC2779828

[B47] Spitzer B, Haegens S (2017) Beyond the status quo: a role for beta oscillations in endogenous content (re)activation. eNeuro 4:ENEURO.0170-17.2017. 10.1523/ENEURO.0170-17.2017 PMC5539431 28785729

[B48] Surwillo WW (1961) Frequency of the “alpha” rhythm, reaction time and age. Nature 191:823–824. 10.1038/191823a0

[B49] Thomson RE, Emery WJ (2014) Time series analysis methods. In: Data analysis methods in physical oceanography, Chapt 5, Ed 3 (Thomson RE, Emery WJ, eds), pp 425–591. Boston: Elsevier.

[B50] Van Veen BD, van Drongelen W, Yuchtman M, Suzuki A (1997) Localization of brain electrical activity via linearly constrained minimum variance spatial filtering. IEEE Trans Biomed Eng 44:867–880. 10.1109/10.623056 9282479

[B51] Vinck M, van Wingerden M, Womelsdorf T, Fries P, Pennartz CMA (2010) The pairwise phase consistency: a bias-free measure of rhythmic neuronal synchronization. Neuroimage 51:112–122. 10.1016/j.neuroimage.2010.01.073 20114076

[B52] Weiss S, Mueller HM (2012) “Too many betas do not spoil the broth”: the role of beta brain oscillations in language processing. Front Psychol 3:201. 10.3389/fpsyg.2012.00201 22737138 PMC3382410

[B53] Weisz N, Hartmann T, Müller N, Lorenz I, Obleser J (2011) Alpha rhythms in audition: cognitive and clinical perspectives. Front Psychol 2:73. 10.3389/fpsyg.2011.00073 PMC3110491 21687444

[B54] Weisz N, Müller N, Jatzev S, Bertrand O (2014) Oscillatory alpha modulations in right auditory regions reflect the validity of acoustic cues in an auditory spatial attention task. Cereb Cortex 24:2579–2590. 10.1093/cercor/bht113 23645711

[B55] Wilsch A, Mercier MR, Obleser J, Schroeder CE, Haegens S (2020) Spatial attention and temporal expectation exert differential effects on visual and auditory discrimination. J Cogn Neurosci 32:1562–1576. 10.1162/jocn_a_01567 32319865 PMC8078477

[B56] Wimmer K, Ramon M, Pasternak T, Compte A (2016) Transitions between multiband oscillatory patterns characterize memory-guided perceptual decisions in prefrontal circuits. J Neurosci 36:489–505. 10.1523/JNEUROSCI.3678-15.2016 26758840 PMC4710772

[B57] Wong YT, Fabiszak MM, Novikov Y, Daw ND, Pesaran B (2016) Coherent neuronal ensembles are rapidly recruited when making a look-reach decision. Nat Neurosci 19:327–334. 10.1038/nn.4210 26752158 PMC4731255

[B58] Wutz A, Melcher D, Samaha J (2018) Frequency modulation of neural oscillations according to visual task demands. Proc Natl Acad Sci U S A 115:1346–1351. 10.1073/pnas.1713318115 29358390 PMC5819398

